# Adult Rectal Prolapse; Two Cases and a Contrast

**Published:** 1917-12

**Authors:** Ralph W. Jackson

**Affiliations:** Fall River, Mass.


### ADULT RECTAL PROLAPSE; TWO CASES AND A CONTRAST.

**By Ralph W. Jackson, M.D., F.A.C.S., Fall River, Mass.**

Too few papers have appeared from the pens of proctologists on the major types of rectal prolapse. The subject deserves more attention. Two sharply contrasting cases bring out the writer's views of the therapy. The first was operated four times; extensive regional cauterization resulting in recurrence; posterior rectopexy resulting in recurrence, amputation, resulting satisfactorily as regards anal prolapse, but leaving a very large posterior vaginal hernia, which was promptly cured by cul de sac closure with sigmoid-uteroventral wall suspension. Then second, and less favorable, case was promptly cured by one operation identical with the final operation in the first case. The pelvic floor as a support depends on the sufficiency of four factors. The pelvic fascia is variable in strength and attachments, varying the depth of the peritoneal cul de sac. The strength of the levators orifices may be guarded by sphincters, peri-vulvar muscles, a perineum and levators which are imperfect through traumam or atrophy. An amount of adipose padding about these structures is essential but often lacking. The rectum has imperfect support above the pelvic floor and its anterior wall none at all. Prolapse begins here from external pressure, or begins below as anal protrusion, but eventually the prolapse contains a hernial cul de sac. Regional cauterization is inadequate to meet any such faults. Posterior rectopexy does not support the primarily offending part. Amputation removes the troublesome part but does not correct the faulty factors which may allow recurrence per annum or per vaginam. Cul de sac closure, though a difficult operation, goes far toward such correction, and suspension helps. The writer should summarizez, that the operation of first choice for these very troublesome prolapses is cul de sac closure through the obdomen, plus some form of suspension, to be followed and supplemented, if need be, later by amputation or perhaps some plastic work on the elements of the pelvic floor.

